# Supplementary material for: A multiplex method for rapidly identifying viral protease inhibitors
Source: Mol Syst Biol. 2025 Jan 6;21(2):158–72. doi: 10.1038/s44320-024-00082-1 (PMC11790949; doi:10.1038/s44320-024-00082-1)
Supplement: Supplementary file 1 — Appendix [file 44320_2024_82_MOESM1_ESM.pdf]

Appendix

A multiplex method for rapidly identifying viral protease inhibitors

Seo Jung Hong[1,19], Samuel J. Resnick[1,2,3,19], Sho Iketani[4,5,19], Ji Won Cha[6], Benjamin Alexander Albert[6,7], Christopher T. Fazekas[6], Ching-Wen Chang[5,8], Hengrui Liu[9], Shlomi Dagan[10], Michael R. Abagyan[11], Pavla Fajtová[11], Bruce Culbertson[2,12], Brooklyn Brace[1], Eswar R. Reddem[13,14], Farhad Forouhar[15], J. Fraser Glickman[10], James M. Balkovec[8], Brent R. Stockwell[9, 15], Lawrence Shapiro[4,13,14], Anthony J. O'Donoghue[11], Yosef Sabo[4,5], Joel S. Freundlich[16,17], David D. Ho[4,5,18], Alejandro Chavez[1,6,*]

[1] Department of Pathology and Cell Biology, Columbia University Vagelos College of Physicians and Surgeons, New York, NY, 10032, USA
[2] Medical Scientist Training Program, Columbia University Irving Medical Center, New York, NY, 10032, USA
[3] Department of Medicine, Columbia University Irving Medical Center, New York, NY, 10032, USA
[4] Aaron Diamond AIDS Research Center, Columbia University Vagelos College of Physicians and Surgeons, New York, NY, 10032, USA
[5] Division of Infectious Diseases, Department of Medicine, Columbia University Vagelos College of Physicians and Surgeons, New York, NY, 10032, USA
[6] Department of Pediatrics, University of California San Diego, La Jolla, CA, 92123, USA
[7] Department of Cellular and Molecular Medicine, University of California San Diego, La Jolla, CA, 92093, USA
[8] Center for Discovery and Innovation, Hackensack Meridian Health, Nutley, NJ, 07110, USA
[9] Department of Biological Sciences, Department of Chemistry, and Department of Pathology and Cell Biology, Columbia University, New York, NY, 10032, USA
[10] Fisher Drug Discovery Resource Center, The Rockefeller University, New York, NY, 10065, USA
[11] Skaggs School of Pharmacy and Pharmaceutical Sciences, University of California, San Diego, La Jolla, CA, 92093, USA
[12] Integrated Program in Cellular, Molecular, and Biomedical Studies, Columbia University Vagelos College of Physicians and Surgeons, New York, NY, 10032, USA
[13] Department of Biochemistry and Molecular Biophysics, Columbia University, New York, NY, 10032, USA
[14] Zuckerman Mind Brain Behavior Institute, Columbia University, New York, NY, 10027, USA
[15] Department of Pathology and Cell Biology and Columbia University Digestive and Liver Disease Research Center, Vagelos College of Physicians and Surgeons, Columbia University Irving Medical Center, New York, 10032, USA
[16] Department of Pharmacology, Physiology, and Neuroscience, Rutgers University – New Jersey Medical School, Newark, NJ, 07103, USA
[17] Department of Medicine, Center for Emerging and Re-emerging Pathogens, Rutgers University – New Jersey Medical School, Newark, NJ, 07103, USA

[18] Department of Microbiology and Immunology, Columbia University Vagelos College of Physicians and Surgeons, New York, NY, 10032, USA
[19] These authors contributed equally.
* Corresponding author

Table of Contents

Appendix Figure S1. Growth defect in yeast caused by catalytic activity of viral proteases. 6

Appendix Figure S2. Tuning toxicity of viral proteases 7

Appendix Figure S3. Yeast as biosensor for kinase activity. 8

Appendix Figure S4. DNA-barcoded viral protease models as biosensors for inhibitory compounds. 9

Appendix Figure S5. Distribution of magratios and well reads. 10

Appendix Figure S6. Validation of broad-acting compounds identified from multiplex drug screening in individual yeast models. 11

Appendix Figure S7. Validation of selective compounds identified from multiplex drug screening in individual yeast models. 13

Appendix Figure S8. Route used for synthesis of chromen-2-one hits. 15

Appendix Figure S9. Time-dependent inhibition of SARS-CoV-2 3CL protease. 16

Appendix Figure S10. Mechanism of CB6778425 binding to SARS-CoV-2 3CL protease. 17

Appendix Figure S11. Route used for synthesis of NSC287495. 18

Appendix Figure S12. Activity of NSC287495 derivatives against SARS-CoV PLP and Bat-SL-CoVZC45 PLP in yeast models. 19

Appendix Figure S13. Synthetic route to active pyridine methylcarbamate analogs of NSC287495. 20

Appendix Figure S14. Inactive pyridine compounds synthesis and activity. 21

Appendix Figure S15. Time-dependent inhibition of SARS-CoV-2 PLP. 22

Appendix Figure S16. SARS CoV-2 virus inhibition assay 23

Appendix Figure S17. Cytotoxicity assays for coronavirus PLP inhibitor analogs. 24

Appendix Figure S18. GRL0617 against SARS-CoV PLP and SARS-CoV-2 PLP in yeast models. 25

Appendix Figure S19. Multiplexing capacity of the screen. 26

Appendix Figure S20. Dataset exhibits homoscedasticity. 27
Appendix Supplementary Methods 28
Appendix Supplementary Note S1 34
Appendix Supplementary Note S2 35

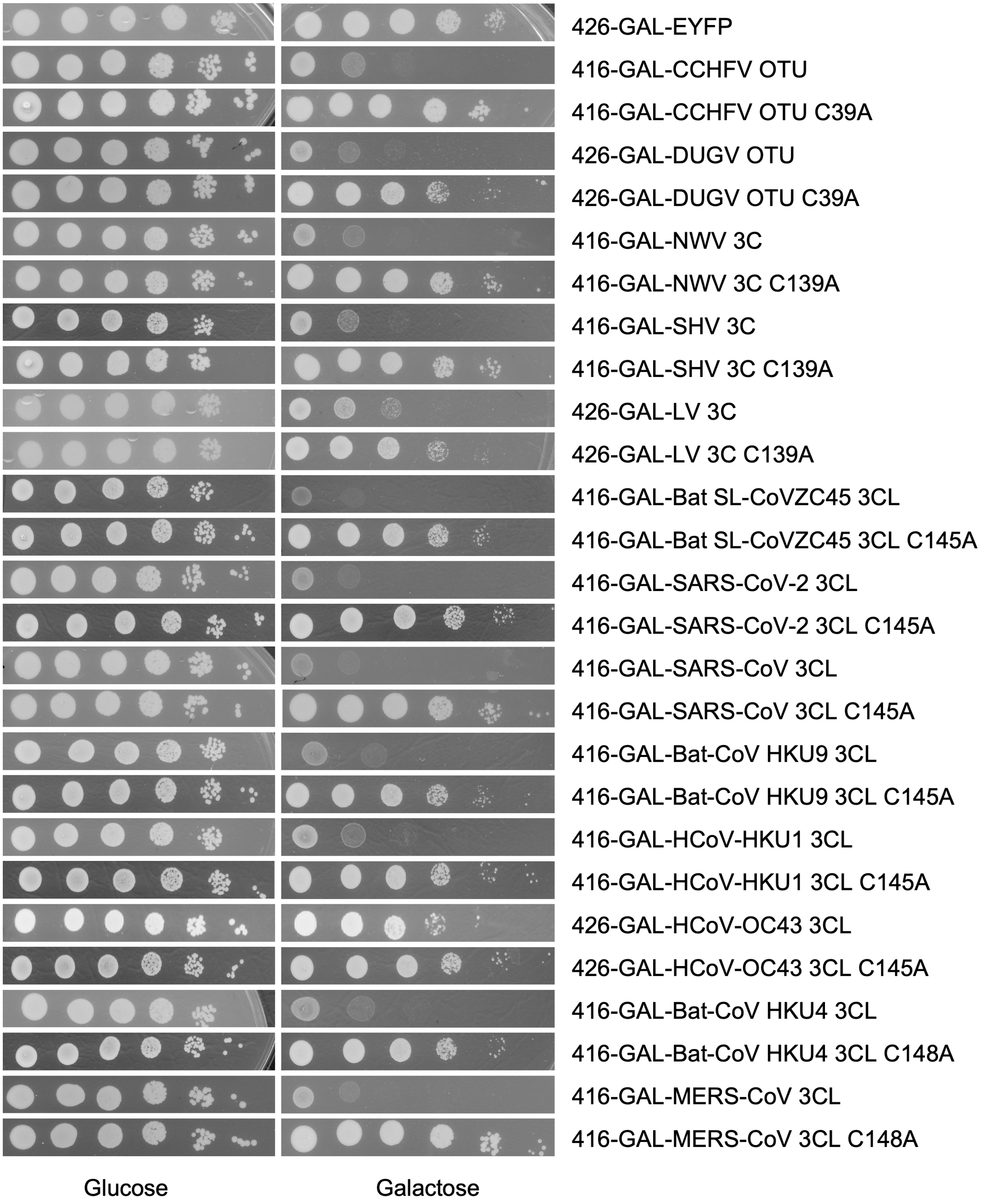
426-GAL-EYFP
416-GAL-CCHFV OTU
416-GAL-CCHFV OTU C39A
426-GAL-DUGV OTU
426-GAL-DUGV OTU C39A
416-GAL-NWV 3C
416-GAL-NWV 3C C139A
416-GAL-SHV 3C
416-GAL-SHV 3C C139A
426-GAL-LV 3C
426-GAL-LV 3C C139A
416-GAL-Bat SL-CoVZC45 3CL
416-GAL-Bat SL-CoVZC45 3CL C145A
416-GAL-SARS-CoV-2 3CL
416-GAL-SARS-CoV-2 3CL C145A
416-GAL-SARS-CoV 3CL
416-GAL-SARS-CoV 3CL C145A
416-GAL-Bat-CoV HKU9 3CL
416-GAL-Bat-CoV HKU9 3CL C145A
416-GAL-HCoV-HKU1 3CL
416-GAL-HCoV-HKU1 3CL C145A
426-GAL-HCoV-OC43 3CL
426-GAL-HCoV-OC43 3CL C145A
416-GAL-Bat-CoV HKU4 3CL
416-GAL-Bat-CoV HKU4 3CL C148A
416-GAL-MERS-CoV 3CL
416-GAL-MERS-CoV 3CL C148A
Glucose
Galactose

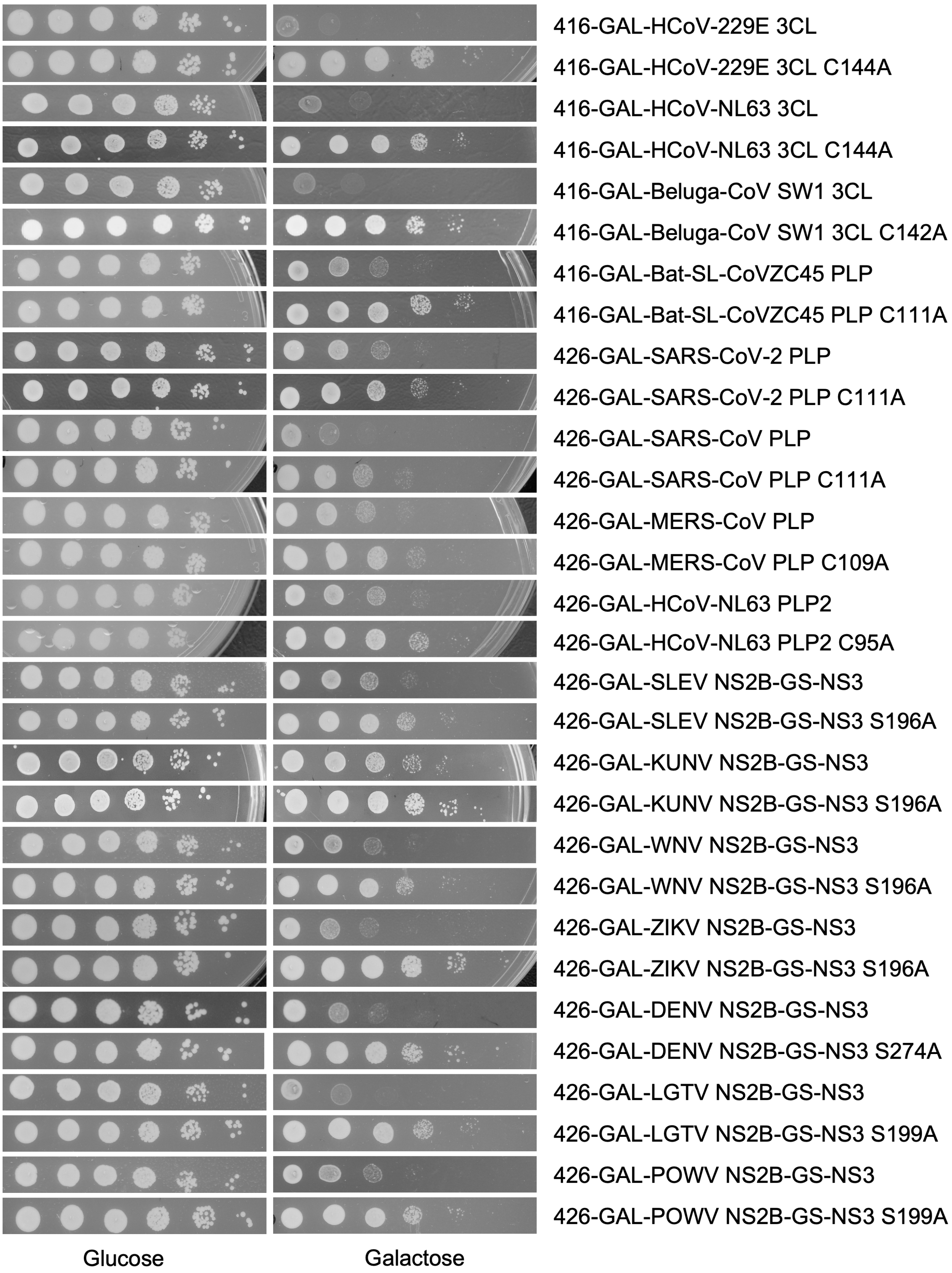
416-GAL-HCoV-229E 3CL
416-GAL-HCoV-229E 3CL C144A
416-GAL-HCoV-NL63 3CL
416-GAL-HCoV-NL63 3CL C144A
416-GAL-Beluga-CoV SW1 3CL
416-GAL-Beluga-CoV SW1 3CL C142A
416-GAL-Bat-SL-CoVZC45 PLP
416-GAL-Bat-SL-CoVZC45 PLP C111A
426-GAL-SARS-CoV-2 PLP
426-GAL-SARS-CoV-2 PLP C111A
426-GAL-SARS-CoV PLP
426-GAL-SARS-CoV PLP C111A
426-GAL-MERS-CoV PLP
426-GAL-MERS-CoV PLP C109A
426-GAL-HCoV-NL63 PLP2
426-GAL-HCoV-NL63 PLP2 C95A
426-GAL-SLEV NS2B-GS-NS3
426-GAL-SLEV NS2B-GS-NS3 S196A
426-GAL-KUNV NS2B-GS-NS3
426-GAL-KUNV NS2B-GS-NS3 S196A
426-GAL-WNV NS2B-GS-NS3
426-GAL-WNV NS2B-GS-NS3 S196A
426-GAL-ZIKV NS2B-GS-NS3
426-GAL-ZIKV NS2B-GS-NS3 S196A
426-GAL-DENV NS2B-GS-NS3
426-GAL-DENV NS2B-GS-NS3 S274A
426-GAL-LGTV NS2B-GS-NS3
426-GAL-LGTV NS2B-GS-NS3 S199A
426-GAL-POWV NS2B-GS-NS3
426-GAL-POWV NS2B-GS-NS3 S199A
Glucose
Galactose

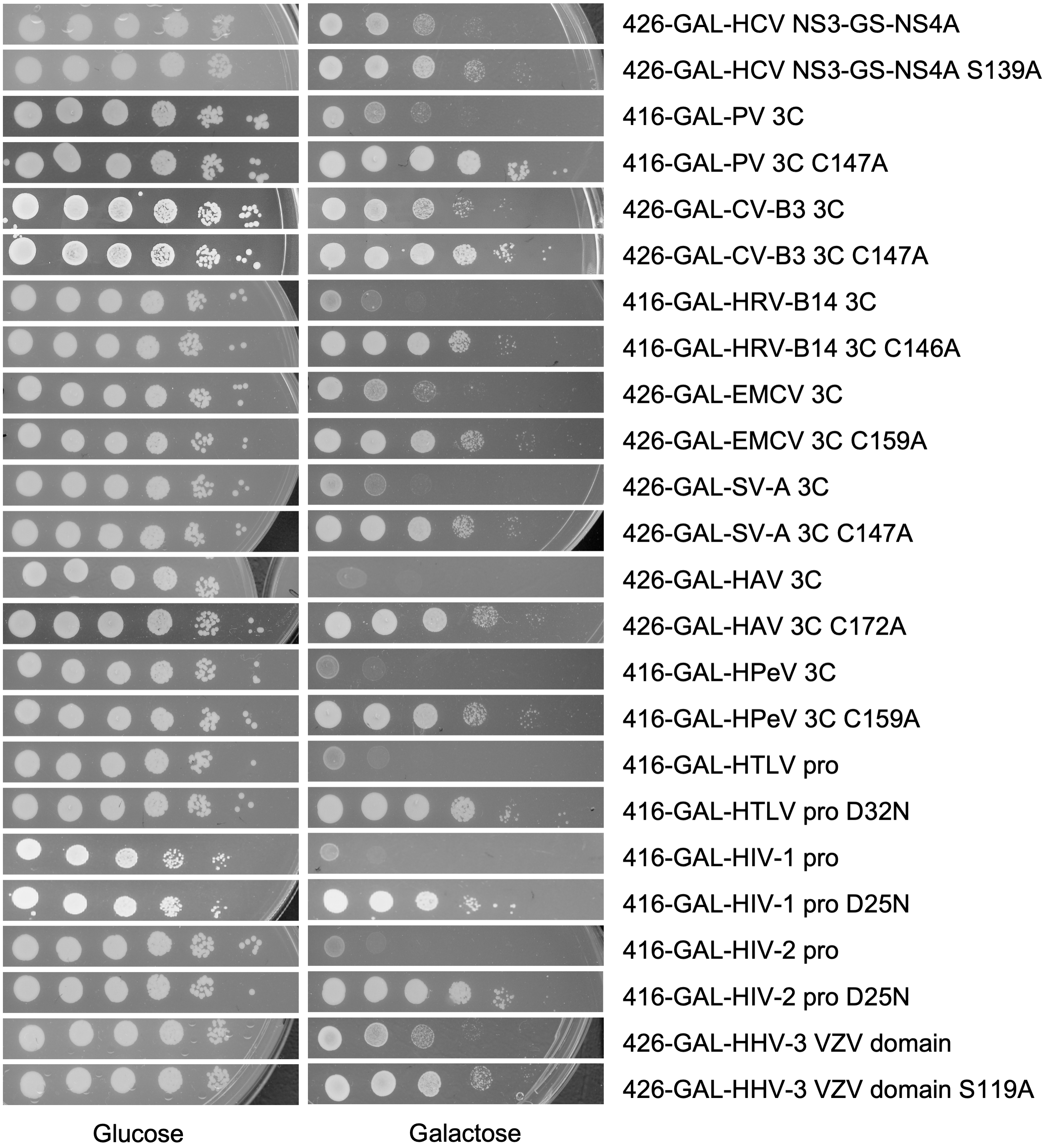

Appendix Figure S1. Growth defect in yeast caused by catalytic activity of viral proteases.

Spot assays representing 10x serial dilution of cells plated to non-inducing (glucose) and inducing (galactose) media are shown for each protease expressing yeast strain. Expression of catalytically deficient mutants of the 40 viral proteases in yeast show an abrogation of the growth defect upon galactose induction as compared to strains expressing the wild type proteins.

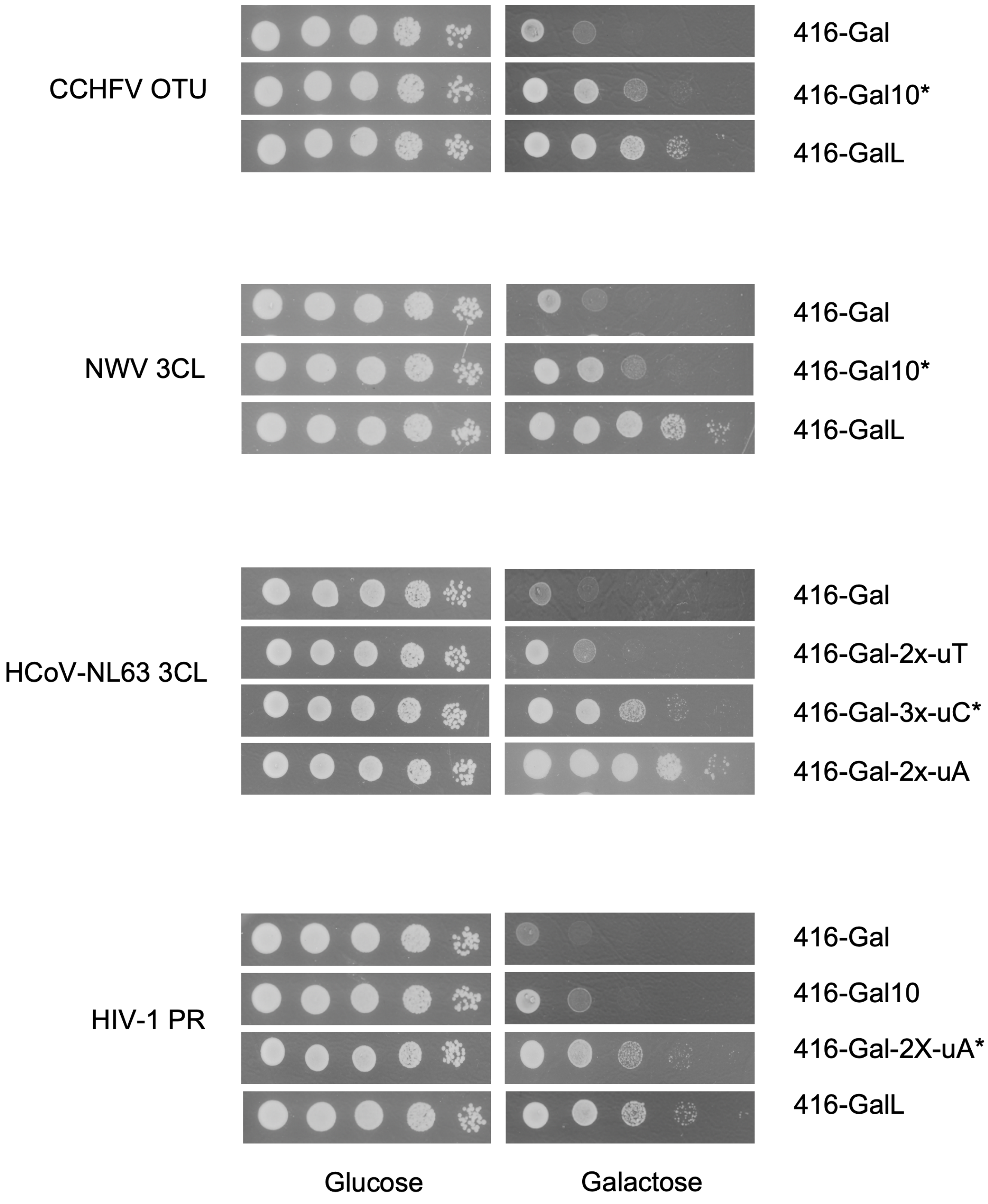

Appendix Figure S2. Tuning toxicity of viral proteases.
Spot assays representing 10x serial dilution of cells plated to non-inducing (glucose) and inducing (galactose) media are shown for each protease expressing yeast strain. Representative viral protease models shown when expressed from different galactose inducible promoters. Selected variants used in the screening pool marked with asterisk.

A

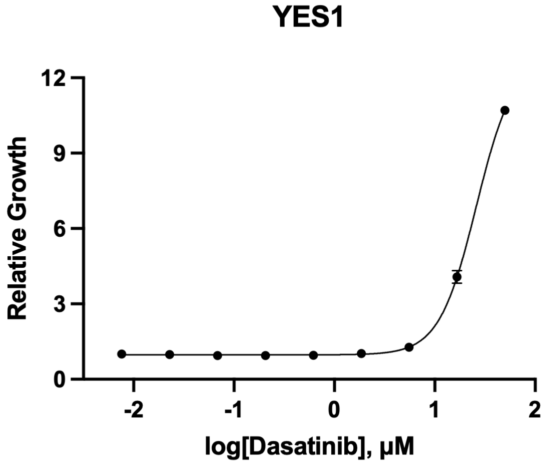

B

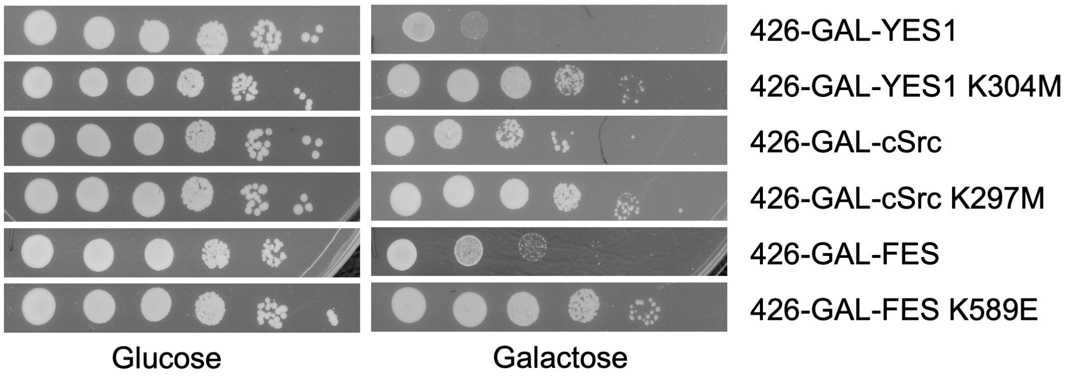

Appendix Figure S3. Yeast as biosensor for kinase activity.
(**A**) Dose-dependent rescue of growth in *S. cerevisiae* expressing human kinase, YES1, by kinase inhibitor, dasatinib. Error bars denote mean ± s.d. of three technical replicates. Growth curve was determined by nonlinear regression and the x axis is in log_{10} scale.
(**B**) Spot assays representing 10x serial dilution of cells plated to non-inducing (glucose) and inducing (galactose) media are shown for each kinase expressing yeast strain. Expression of catalytically deficient mutants of the 3 kinases in yeast show an abrogation of the growth defect upon galactose induction as compared to strains expressing the wild type proteins.

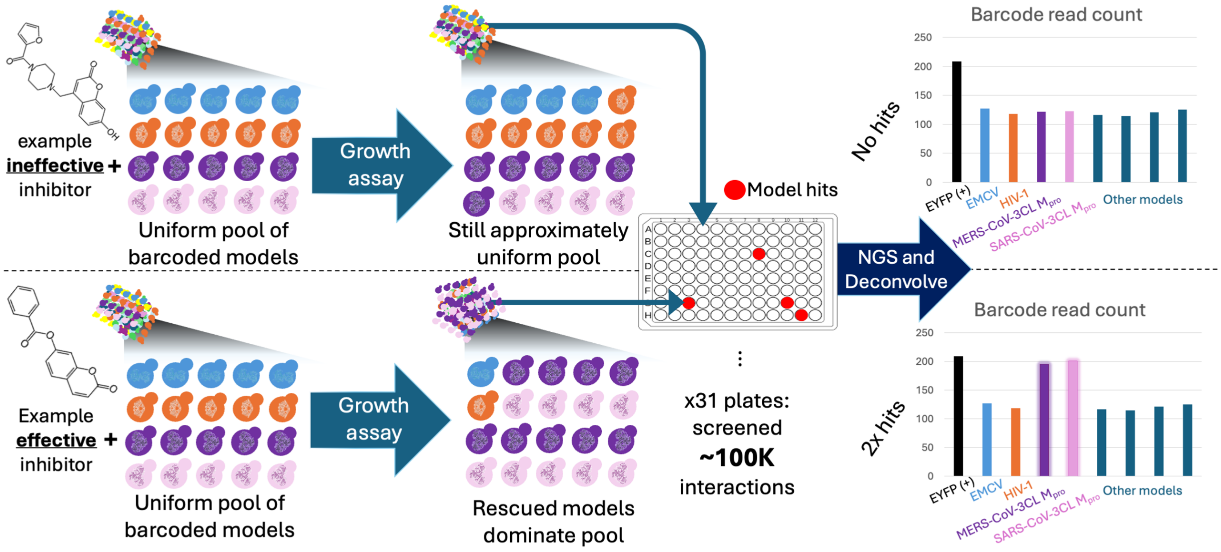

Appendix Figure S4. DNA-barcoded viral protease models as biosensors for inhibitory compounds.
DNA-barcoding enables pooled drug screening of various viral protease and control-expressing yeast strains (i.e., models). Due to the toxicity induced by active viral proteases or orthogonally toxic controls (i.e., kinases) within the yeast, the presence of an inhibitory compound leads to a growth rescue, which can be detected via targeted amplicon sequencing and quantification of barcodes representing each viral protease and control within the pool of models.

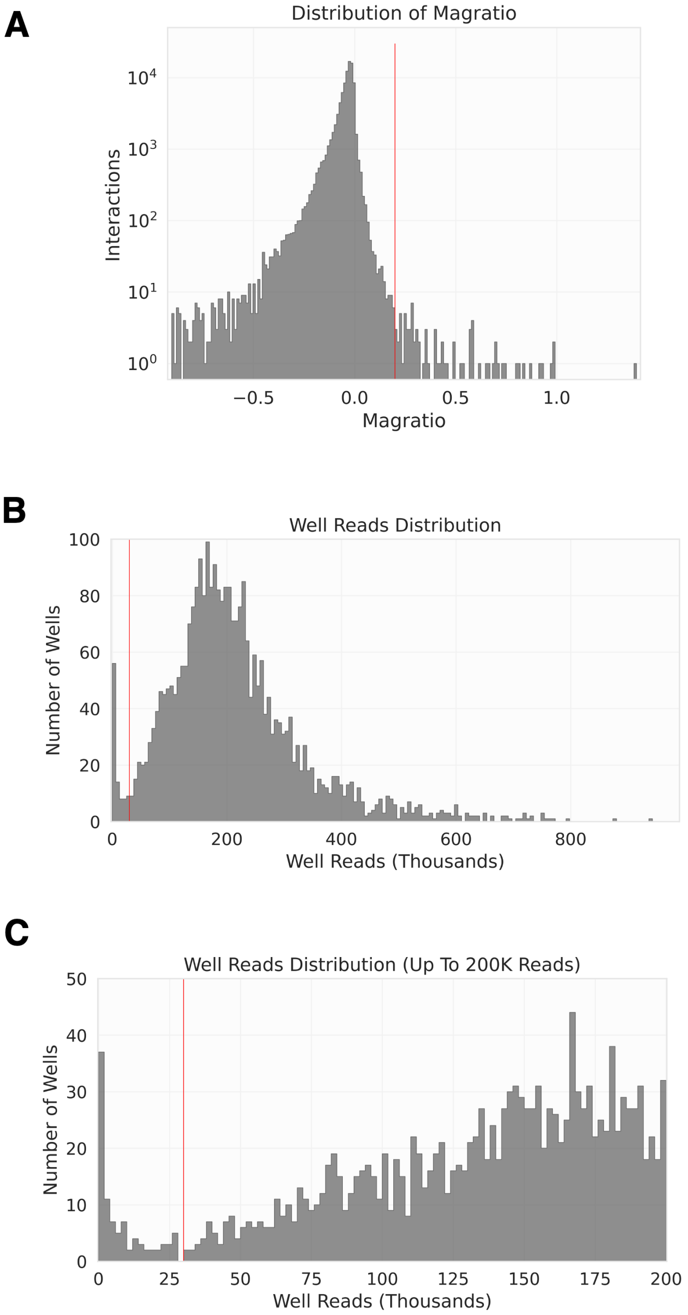

Appendix Figure S5. Distribution of magratios and well reads.
(**A**) Distribution of magratios across the 106,640 total model-compound interactions analyzed. Top 79 interactions with magratios > 0.2 (indicated by red line) were selected for validation in individual yeast models. (**B – C**) Distribution of well reads across all wells analyzed (**B**), and those with up to 200,000 reads (**C**). Wells were filtered by read counts with a minimum threshold set to 30,000 total reads in order to be analyzed.

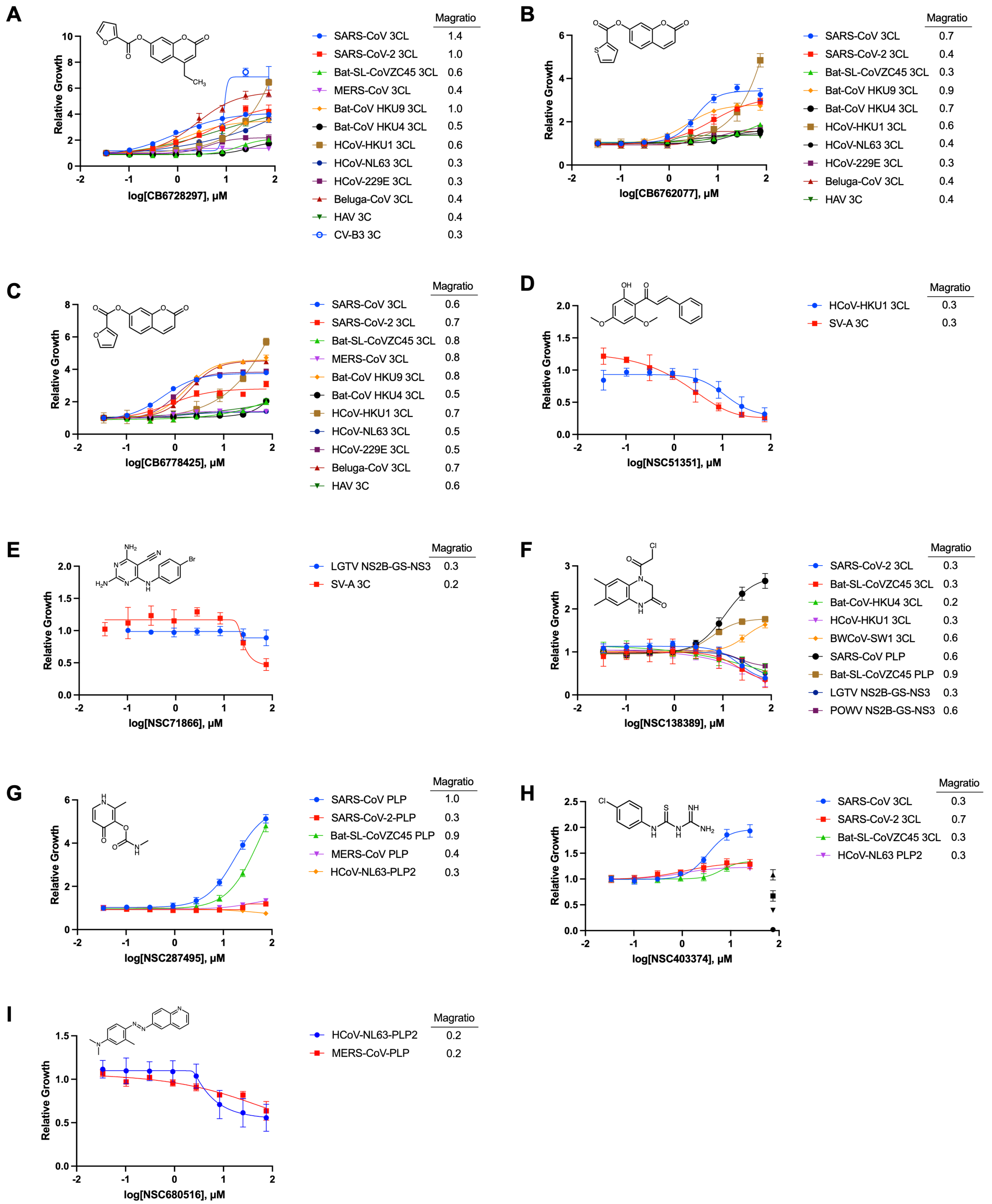

Appendix Figure S6. Validation of broad-acting compounds identified from multiplex drug screening in individual yeast models.

(**A – I**) Dose-response assessment of indicated compounds on the growth of yeast expressing various viral proteases. Assay was conducted with all individually barcoded

strains for each model. Error bars denote mean ± s.d. of four or five biological replicates. Growth curves were determined by nonlinear regression and the x axis is in $\log_{10}$ scale. For NSC403374 (**H**), which led to dose-dependent rescue but induced toxicity at the highest tested concentration (75.0 μM), the final data points (shown in black) were removed for deriving the growth curves.

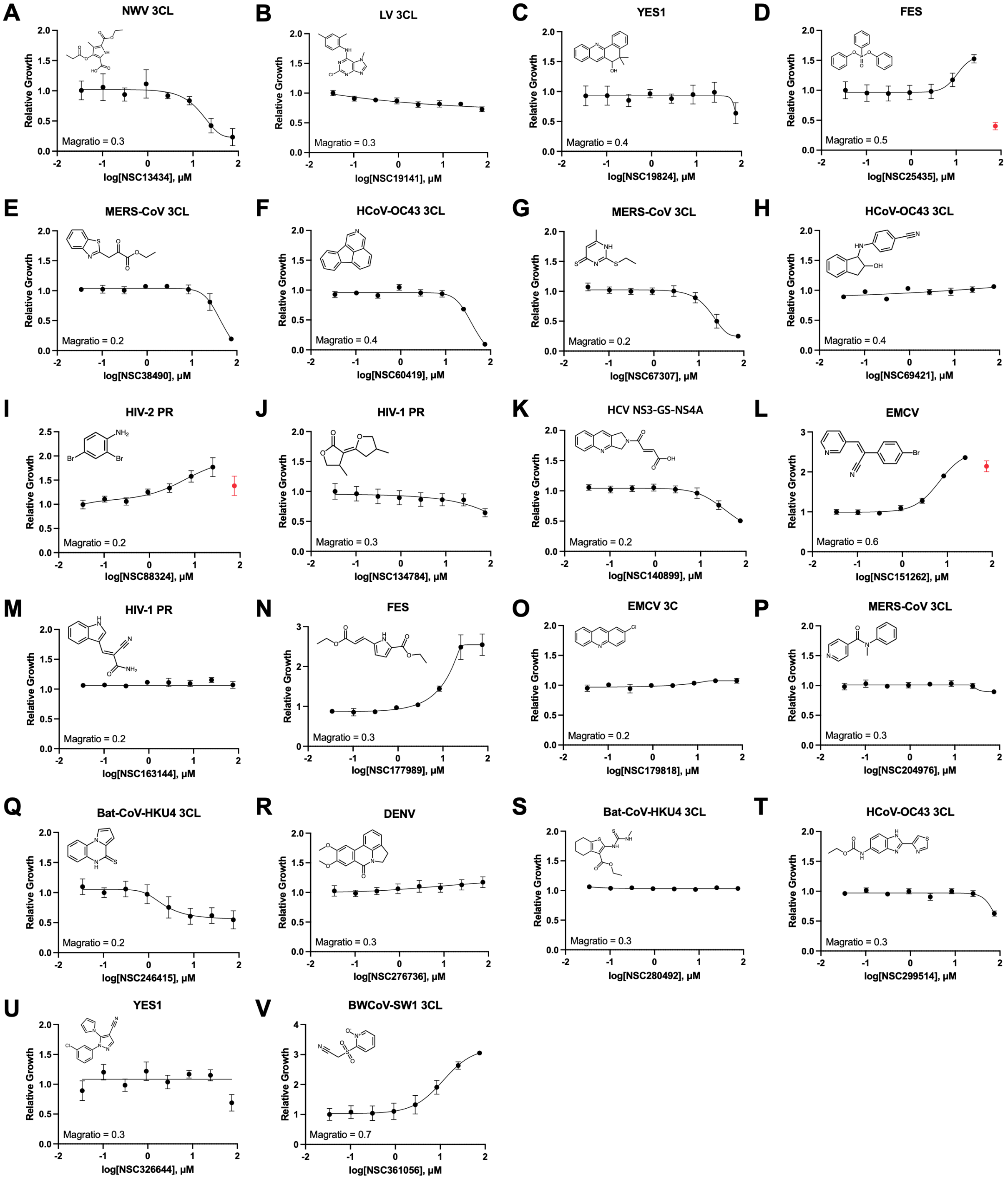

Appendix Figure S7. Validation of selective compounds identified from multiplex drug screening in individual yeast models.

(**A – V**) Dose-response assessment of indicated compounds on the growth of yeast expressing various viral proteases and FES kinase. Assay was conducted with all individually barcoded strains for each model. Error bars denote mean ± s.d. of four or

five biological replicates. Growth curves were determined by nonlinear regression and the x axis is in log_{10} scale. For compounds that led to dose-dependent rescue but induced toxicity at the highest tested concentration (75.0 μM), the final data points (shown in red in panels D, I, and L) were removed for deriving the growth curves.

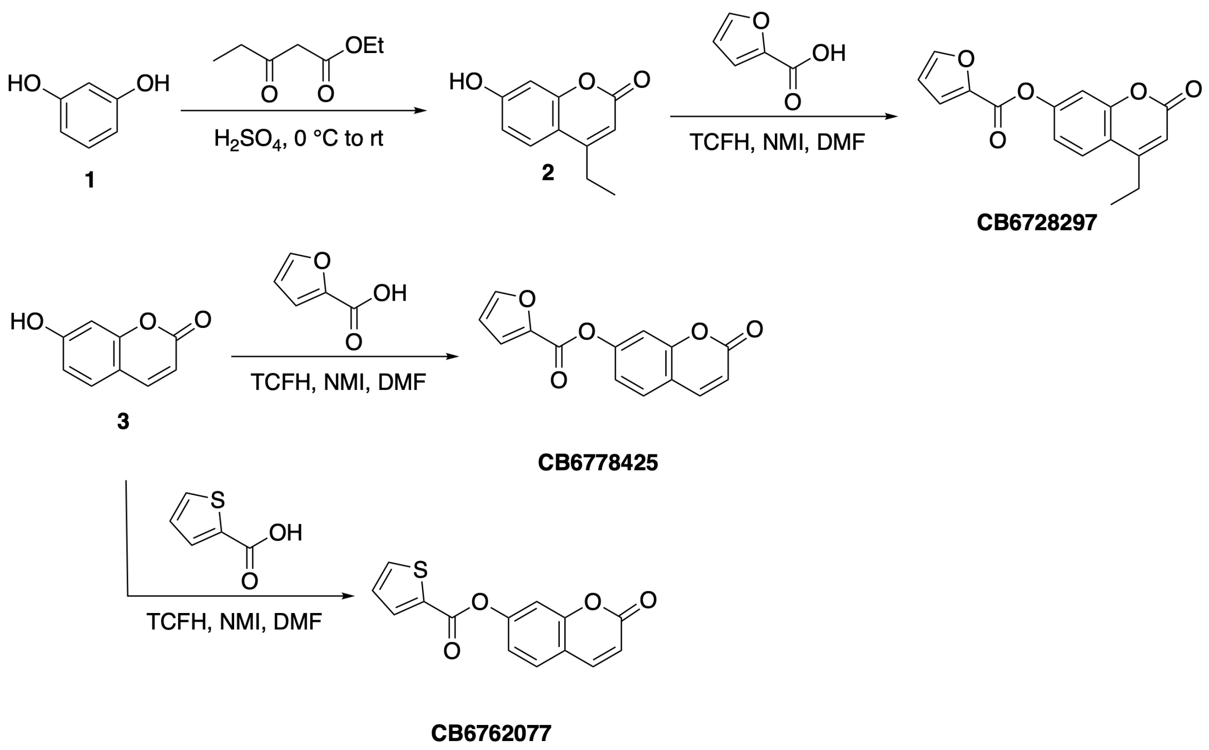

Appendix Figure S8. Route used for synthesis of chromen-2-one hits.

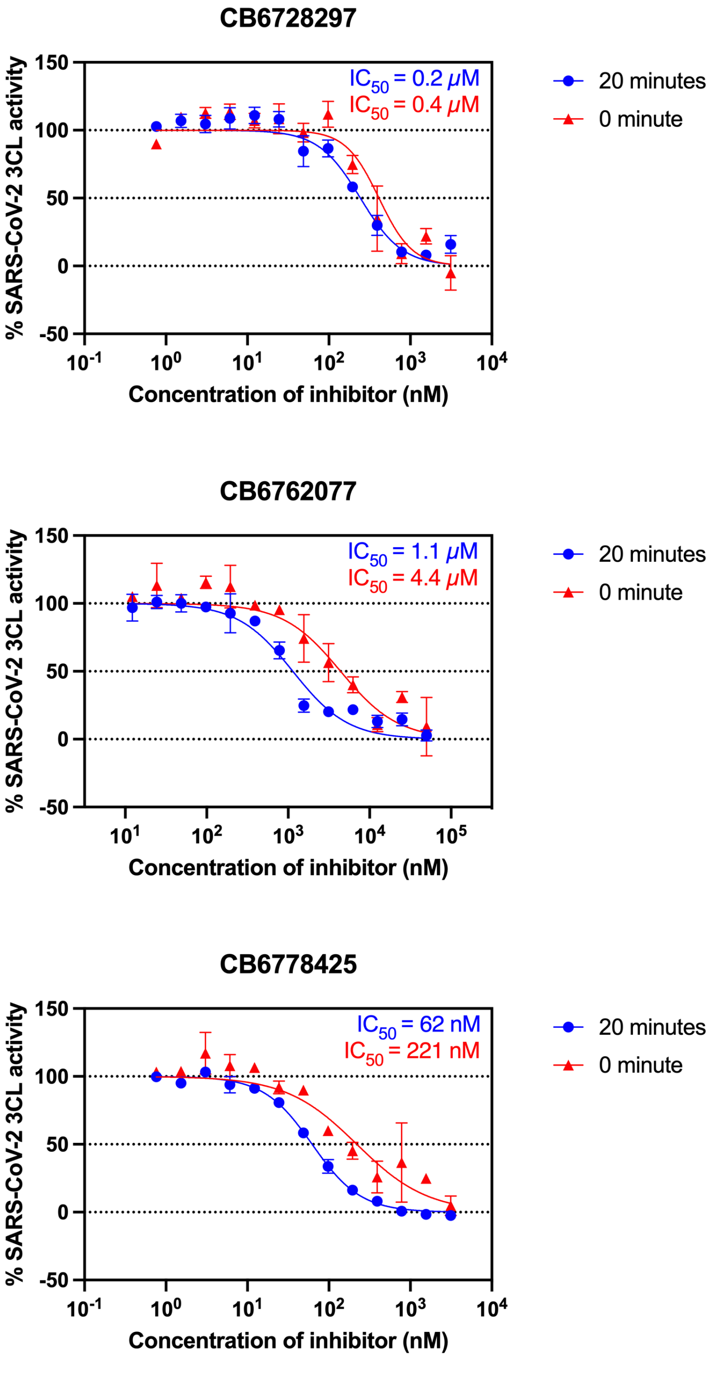

Appendix Figure S9. Time-dependent inhibition of SARS-CoV-2 3CL protease. Inhibition of purified SARS-CoV-2 3CL protease by the chromen-2-one-containing inhibitors upon addition of substrate at 0 and 20 minutes. Error bars denote mean ± s.d. of technical duplicates. IC50 was determined via nonlinear regression. The x axis is in $\log_{10}$ scale.

A

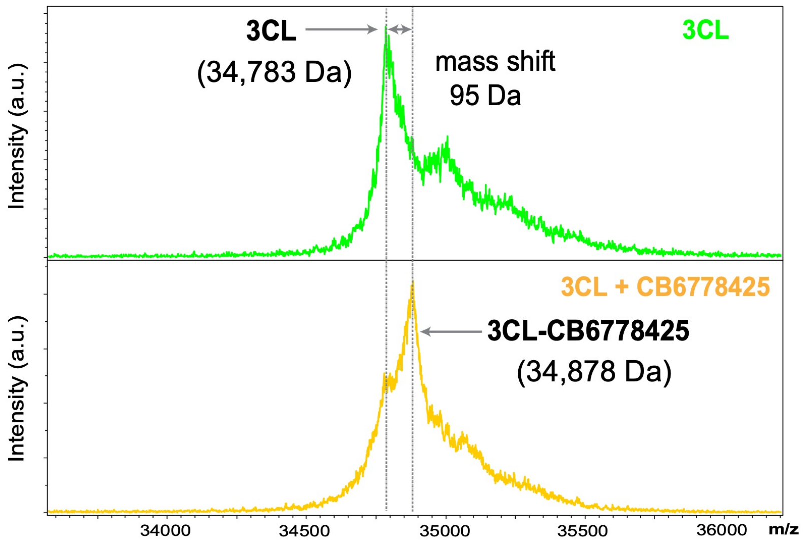

B

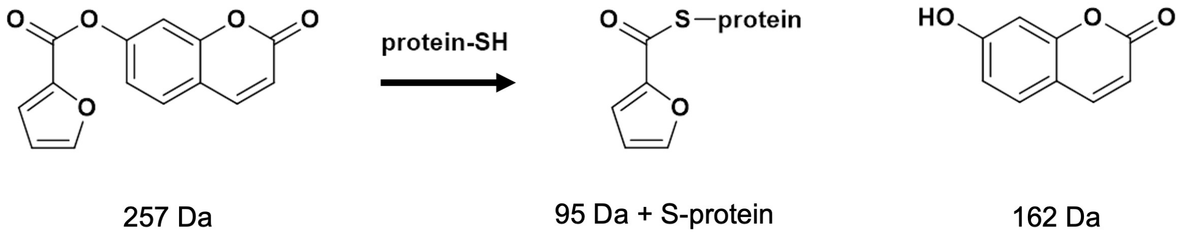

Appendix Figure S10. Mechanism of CB6778425 binding to SARS-CoV-2 3CL protease.

(**A**) MALDI-TOF MS analysis of SARS-CoV-2 3CL protease alone and in complex with CB6778425. (**B**) Predicted mechanism of CB6778425 binding to the Cys145 of the protease via covalent modification.

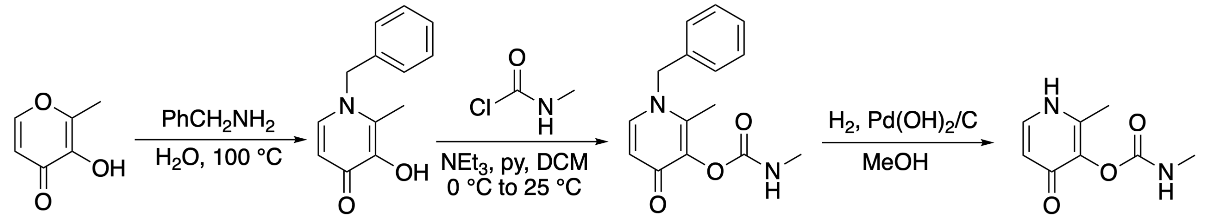

4 **5** **6** **NSC287495**

Appendix Figure S11. Route used for synthesis of NSC287495.

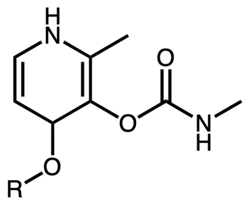

Compound	R	EC_{50} SARS-CoV PLP (μM)	EC_{50} Bat SL-CoVZC45 PLP (μM)
MAVDA-B-116	Me	0.588	0.538
MAVDA-B-190	Et	1.72	0.843
MAVDA-B-217	i-Pr	0.552	0.23
MAVDA-B-219	$CH_2C_6H_{12}$	0.863	0.696
MAVDA-B-201	CH_2Ph	0.66	0.405
NSC287495		22.8	15.6

Appendix Figure S12. Activity of NSC287495 derivatives against SARS-CoV PLP and Bat-SL-CoVZC45 PLP in yeast models.

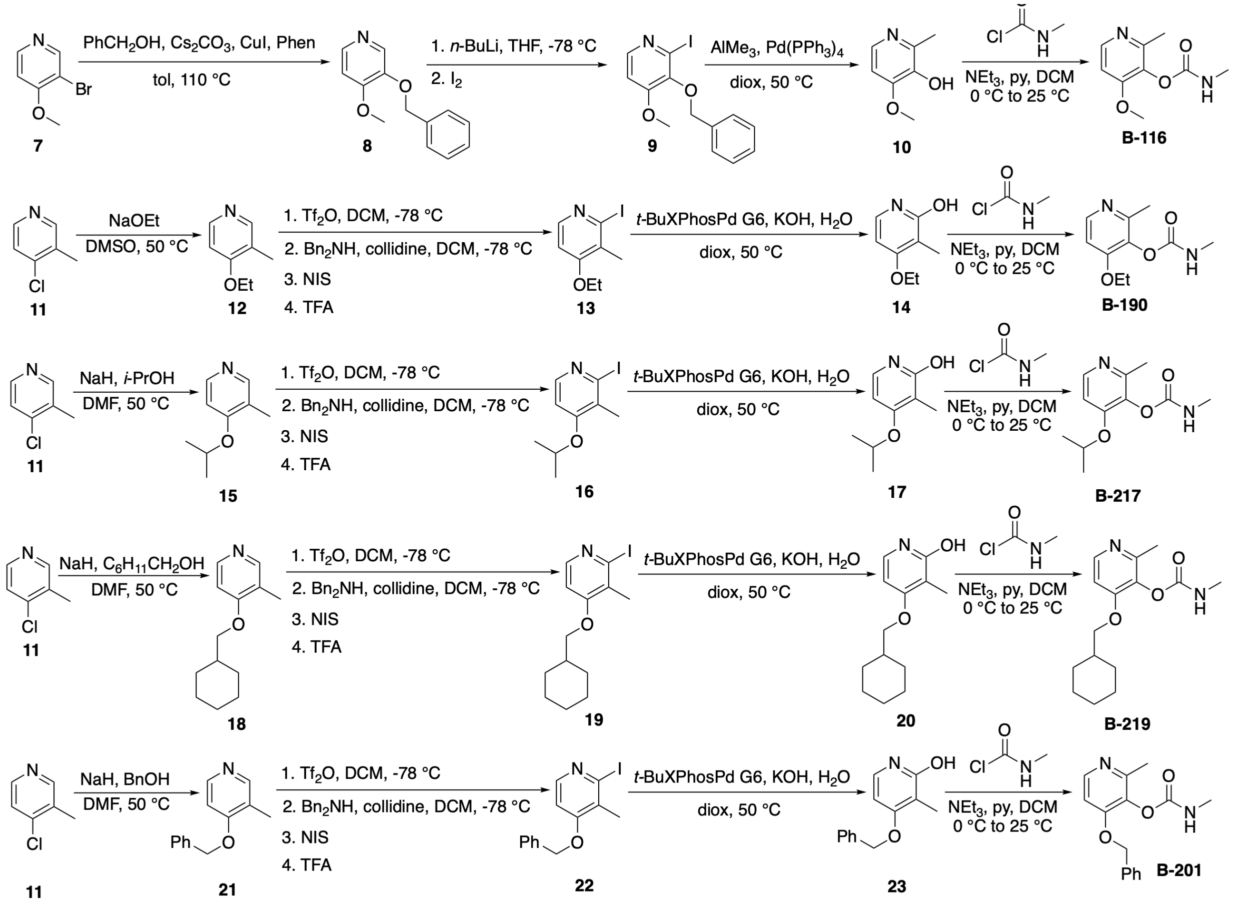

Appendix Figure S13. Synthetic route to active pyridine methylcarbamate analogs of NSC287495.

A

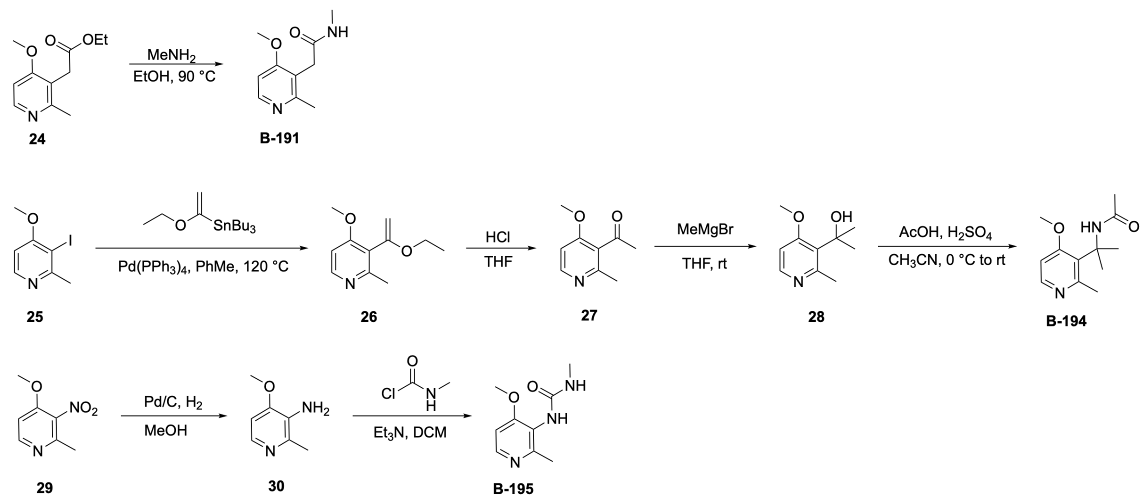

B

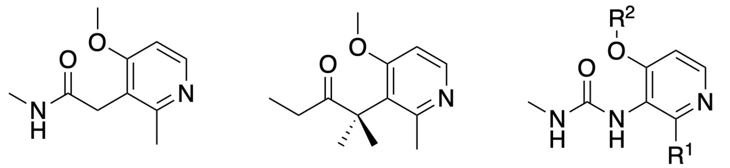

	B-191	B-194	B-195
EC_{50} SARS-CoV PLP (μM)	≥50	≥50	≥20
EC_{50} Bat SL-CoVZC45 PLP2 (μM)	≥20	≥20	≥20

Appendix Figure S14. Inactive pyridine compounds synthesis and activity.
(**A**) Synthetic route to inactive pyridine analogs of NSC287495. (**B**) Replacement of the methylcarbamate in the 4-alkoxy-2-methylpyridin-3-yl methylcarbamate family affords inactive compounds in the yeast assay.

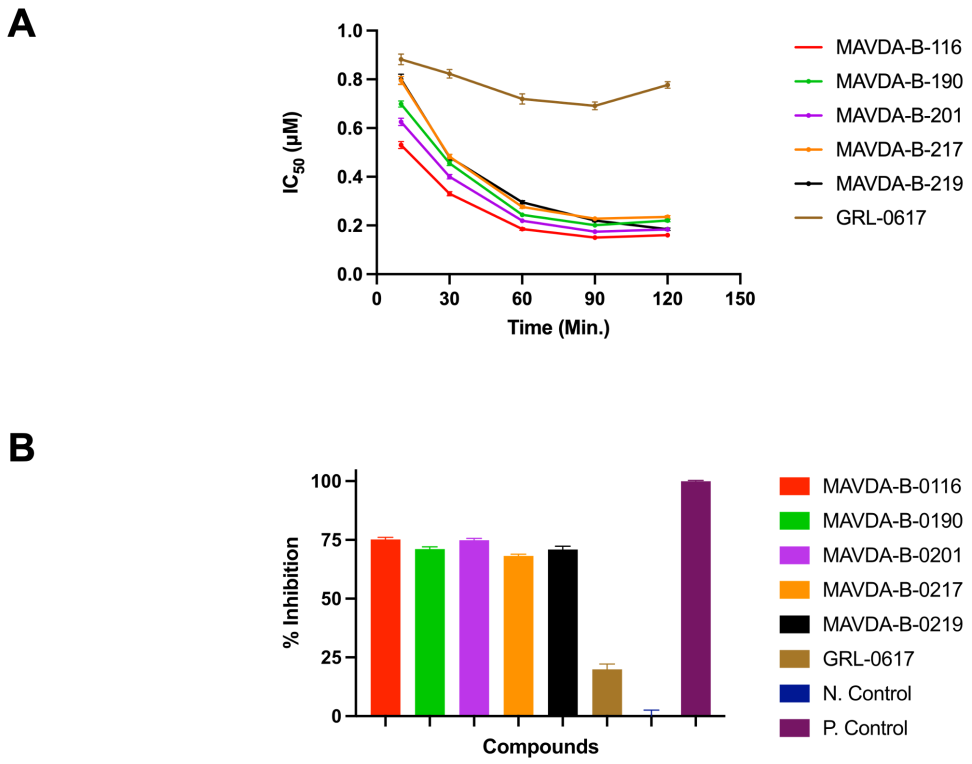

Appendix Figure S15. Time-dependent inhibition of SARS-CoV-2 PLP.
(A) SARS-CoV-2 PLP time-dependent dose-response curve (DRC) assay. IC_{50} values were extracted from the DRC for the inhibition of purified SARS-CoV-2 PLP and plotted against the pre-incubation time. Error bars denote mean±s.d. of five technical replicates. Nonlinear regression was used to determine the IC_{50}s. **(B)** SARS-CoV-2 PLP X100 assay. Inhibition of purified SARS-CoV-2 PLP was measured after pre-incubation of both compound and protein at a high concentration followed by dilution of 100-fold by the substrate. Error bars denote mean± s.e.m. of ten technical replicates. N. Control, or negative control, refers to the assay performed without the addition of a compound. P. Control, or positive control, refers to the assay performed without the enzyme.

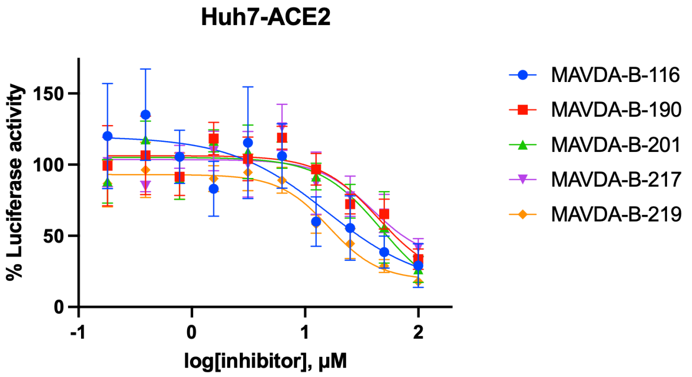

Compound	IC_{50} (μM)
MAVDA-B-116	15.5
MAVDA-B-190	44.2
MAVDA-B-201	47.1
MAVDA-B-217	39.3
MAVDA-B-219	16.3

Appendix Figure S16. SARS CoV-2 virus inhibition assay.
Inhibition of SARS-CoV-2 viral replication in Huh7-ACE2 cells. Error bars denote mean ± s.d. of four technical replicates. Nonlinear regression was used to determine the IC_{50}s and the x axis is in log_{10} scale.

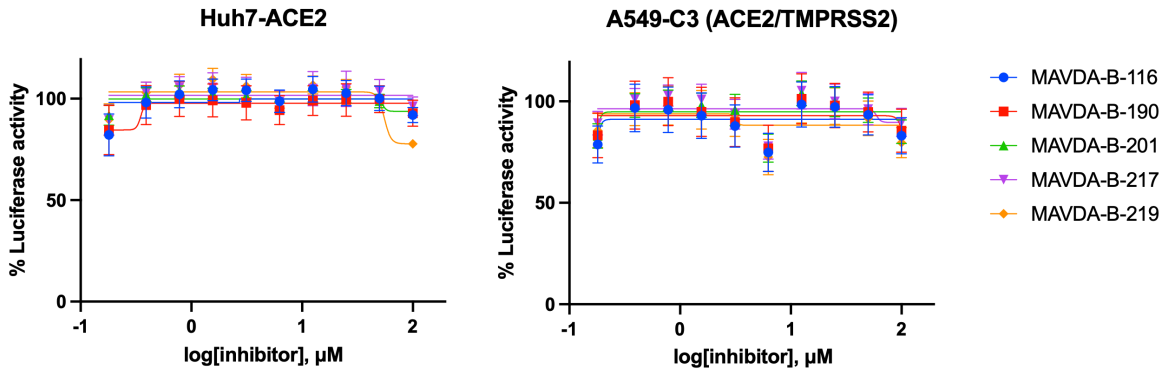

Compound	CC_{50} (μM)
MAVDA-B-116	>100
MAVDA-B-190	>100
MAVDA-B-201	>100
MAVDA-B-217	>100
MAVDA-B-219	>100

Compound	CC_{50} (μM)
MAVDA-B-116	>100
MAVDA-B-190	>100
MAVDA-B-201	>100
MAVDA-B-217	>100
MAVDA-B-219	>100

Appendix Figure S17. Cytotoxicity assays for coronavirus PLP inhibitor analogs. Cytotoxicity of analogs tested in Huh7-ACE2 and A549-C3 cells via detection of luminescence coupled to ATP levels indicative of metabolically active cells. Error bars denote mean ± s.d. of four technical replicates. Nonlinear regression was used to determine the CC_{50}s and the x axis is in log_{10} scale.

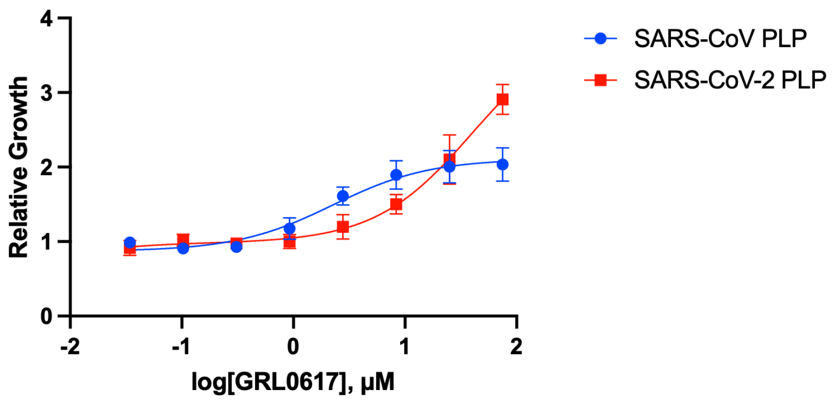

Appendix Figure S18. GRL0617 against SARS-CoV PLP and SARS-CoV-2 PLP in yeast models.

Dose-response assessment of GRL0617 on the growth of yeast expressing the SARS-CoV PLP and SARS-CoV-2 PLP. Assay was conducted with all individually barcoded strains for each model. Error bars denote mean ± s.d. of five biological replicates. Growth curves were determined by nonlinear regression and the x axis is in log_{10} scale.

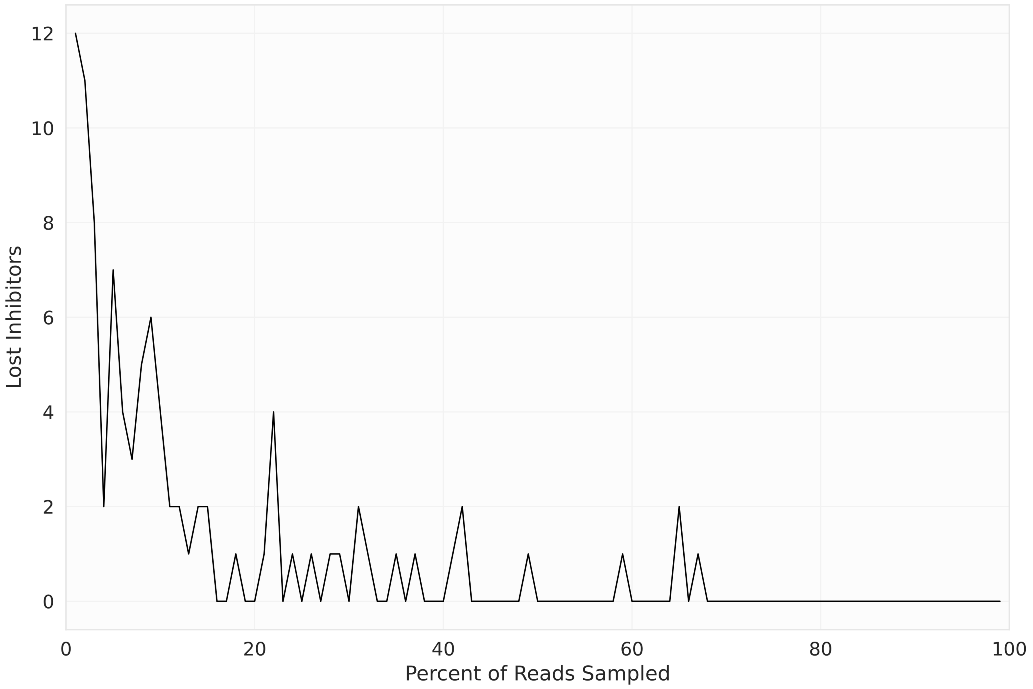

Appendix Figure S19. Multiplexing capacity of the screen.

Progressively lesser sequencing depth was simulated via random subsampling of well reads, retaining only X% of the original reads for X in [1,2,...,99]. Lost inhibitors, resulting from corrupted magratio signals at the reduced sampling depth, is shown to appear at less than ~20% of reads used within the original screening, suggesting capacity to scale the current screen by approximately five-fold without further modification.

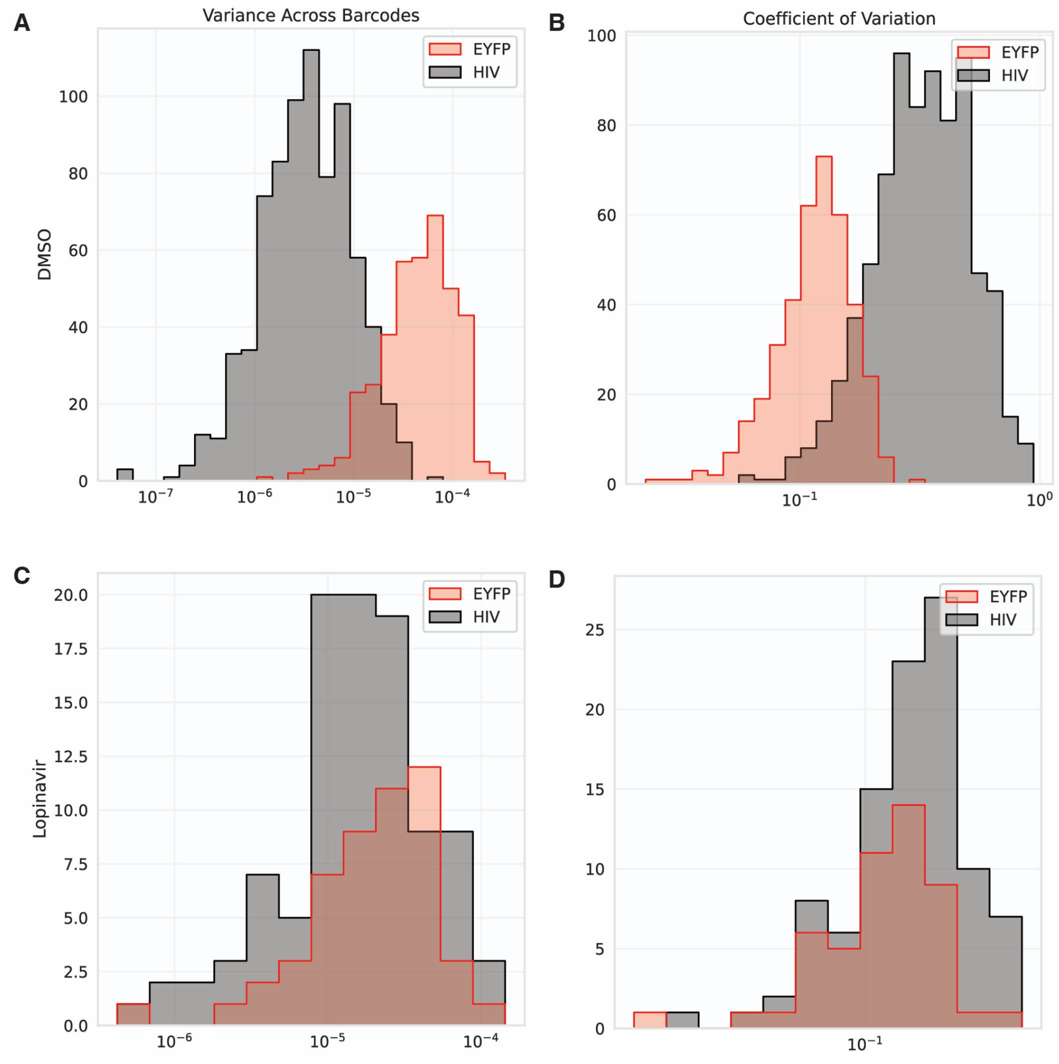

Appendix Figure S20. Dataset exhibits homoscedasticity.

The coefficient of variation (CV) and the variance of well-normalized control gene reads (EYFP, HIV) across barcodes of every plate was plotted for DMSO and Lopinavir wells. In DMSO control wells, we observed that the variance and CV are unimodal with minor skew, as seen in subfigures (A) and (B), suggesting that our negative controls are homoscedastic. Similarly, the positive control well variance and CV in subfigures (C) and (D) are unimodal, but they exhibit stronger skew than the negative controls.

Appendix Supplementary Methods

General Methods. All reagents were purchased from commercial suppliers and used without further purification unless noted otherwise. All chemical reactions occurring solely in an anhydrous organic solvent were carried out under an inert atmosphere of argon or nitrogen unless noted otherwise. Purifications were typically carried out via silica gel column chromatography with a Biotage Isolera One Flash instrument or via preparative HPLC with a Shimadzu LC-MS 2020 mass-directed preparative HPLC system or Shimadzu LC-20AP Preparative HPLC system. HPLC data were attained with a Shimadzu LC-2030C 3D Plus. LC-MS data were collected on a Shimadzu LC20 (binary pump/PDA detector) coupled to a Shimadzu 2020 mass spectrometer. ^{1}H nuclear magnetic resonance spectroscopy (NMR) spectra were recorded on a Bruker AVANCE NEO 400 MHz NMR spectrometer. Chemical shifts, δ, are quoted in parts per million (ppm) relative to TMS and calibrated using residual un-deuterated solvent as an internal reference. The following abbreviations are used to denote the multiplicities and general assignments: s (singlet), d (doublet), t (triplet), q (quartet), dd (doublet of doublets), ddd (doublet of doublet of doublets), dt (doublet of triplets), dq (doublet of quartets), hep (heptet), m (multiplet), pent (pentet), td (triplet of doublets), qd (quartet of doublets), app. (apparent) and br. (broad). Coupling constants, J, are quoted to the nearest 0.1 Hz. All synthesized compounds were >95% pure by HPLC analysis and were characterized by the expected parent ion/s in the MS. Reactions were monitored by thin layer chromatography (TLC) using 0.25 mm silica gel 60 F254 plates or analytical LCMC using one of following methods.

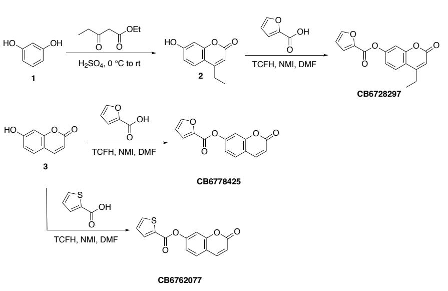

4-ethyl-2-oxo-2H-chromen-7-yl furan-2-carboxylate (CB6728297): Concentrated sulfuric acid (50 mL) was added dropwise to a mixture of resorcinol **1** (10 g, 0.090 mol) in ethyl 3-oxopentanoate **2** (14.4 g, 0.0990 mol) at 0 °C. After addition, the mixture was stirred at 25 °C for 4 h. The reaction mixture was slowly poured into ice water then extracted with EtOAc. The combined organic layers were washed with brine, dried over anhydrous Na_2SO_4, filtered, and concentrated *in vacuo*. The residue was purified by silica gel chromatography (DCM/MeOH, 0% to 10%) to afford 4-ethyl-7-hydroxy-2H-chromen-2-one **2** (9.0 g, 52% yield) as a white solid: LC-MS (ESI) calcd. for $C_{11}H_{11}O_3$ $[M + H]^+$ m/z 191.07, found 190.88.

To a solution of 4-ethyl-7-hydroxy-2H-chromen-2-one **2** (200 mg, 1.05 mmol), furan-2-carboxylic acid **4** (177 mg, 1.57 mmol), NMI (259 mg, 3.15 mmol) in DMF (5 mL) was added TCFH (443 mg, 1.57 mmol) slowly at 0 °C. After addition, the mixture was stirred at 25 °C for 16 h. The reaction was quenched with water and extracted with EtOAc. The combined organic layers were washed with brine, dried over anhydrous Na_2SO_4, filtered, and concentrated *in vacuo*. The residue was purified by prep-HPLC (Gemini 5 µm C_{18} column, 150*21.2 mm, eluting with 20% to 90% MeCN/H_2O containing 0.1% formic acid) to afford the desired product (41.58 mg, 13.91% yield) as a white solid: ^{1}H NMR (400 MHz, DMSO-d_6, ppm) δ 8.14 (dd, J = 1.8, 0.8 Hz, 1 H), 7.93 (d, J = 8.7 Hz, 1 H), 7.63 (dd, J = 3.6, 0.8 Hz, 1 H), 7.46 (d, J = 2.3 Hz, 1 H), 7.34 (dd, J = 8.7, 2.3 Hz,

1 H), 6.83 (dd, *J* = 3.6, 1.7 Hz, 1 H), 6.36 (d, *J* = 1.3 Hz, 1 H), 2.87 (qd, *J* = 7.4, 1.3 Hz, 2 H), 1.26 (t, *J* = 7.4 Hz, 3 H). LC-MS (ESI) calcd. for $C_{16}H_{13}O_5$ $[M + H]^+$ m/z 285.07, found 285.10.

2-oxo-2H-chromen-7-yl furan-2-carboxylate (CB6778425): To a solution of 7-hydroxy-2H-chromen-2-one **1** (200 mg, 1.23 mmol), furan-2-carboxylic acid (207 mg, 1.85 mmol), and NMI (304 mg, 3.70 mmol) in DMF (5 mL) was added TCFH (519 mg, 1.85 mmol) slowly at 0 °C. After addition, the mixture was stirred at 25 °C for 12 h. After competition, the mixture was quenched with water and extracted with EtOAc. The combined organic layers were washed with water and brine, dried over anhydrous Na_2SO_4, and concentrated under vacuum. The residue was purified by prep-HPLC (Gemini 5 μm C_{18} column, 150*21.2 mm, eluting with 20% to 90% MeCN/H_2O containing 0.1% formic acid) to afford the desired product (45 mg, 14% yield) as a white solid: ^{1}H NMR (400 MHz, DMSO-*d6*, ppm) δ 8.16 - 8.13 (m, 1 H), 8.11 (d, J = 9.6 Hz, 1 H) , 7.83 (d, J = 8.4 Hz, 1 H), 7.63 (dd, J = 3.6, 0.8 Hz, 1 H), 7.47 (d, J = 2.0 Hz, 1 H), 7.32 (dd, J = 8.4, 2.0 Hz, 1 H), 6.83 (dd, J = 3.6, 1.6 Hz, 1 H), 6.51 (d, J = 9.6 Hz, 1 H). LC-MS (ESI) calcd. for $C_{14}H_9O_5$ $[M + H]^+$ m/z 257.04, found 257.10.

Compound **CB6762077** was prepared via a similar route and its spectroscopic data are presented below:
2-oxo-2*H*-chromen-7-yl thiophene-2-carboxylate (CB6762077): ^{1}H NMR (400 MHz, DMSO-*d6*, ppm) δ 8.16-8.06 (m, 3 H), 7.83 (d, *J* = 8.5 Hz, 1 H), 7.48 (d, *J* = 2.1 Hz, 1 H), 7.36-7.31 (m, 2 H), 6.51 (d, *J* = 9.6 Hz, 1 H). LC-MS (ESI) calcd. for $C_{14}H_9O_4S$ $[M + H]^+$ m/z 273.01, found 273.10.

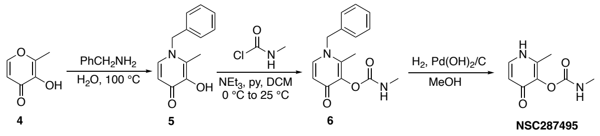

2-methyl-4-oxo-1,4-dihydropyridin-3-yl methylcarbamate (NSC287495): A solution of 3-hydroxy-2-methyl-4H-pyran-4-one **4** (2.000 g, 15.87 mmol) and phenylmethanamine (3.400 g, 31.74 mmol) in H_2O (10 mL) was heated in microwave reactor at 110 °C for 2 h. Upon the reaction completed, the reaction was extracted with EtOAc (100 mL × 3). The combined organic layers were washed with brine, dried over anhydrous Na_2SO_4, filtered, and concentrated *in vacuo*. The residue was purification by silica gel chromatography (DCM/MeOH, 0% to 20%) to afford the desired product **5** (1.46 g, 43.0% yield): ^{1}H NMR (400 MHz, DMSO-*d6*, ppm) δ 7.75 (d, *J* = 7.3 Hz, 1 H), 7.38 (t, *J* = 7.4 Hz, 2 H), 7.32 (d, *J* = 7.3 Hz, 1 H), 7.07 (d, *J* = 7.3 Hz, 2 H), 6.19 (d, *J* = 7.3 Hz, 1 H), 5.25 (s, 2 H), 2.12 (s, 3 H).

To a solution of 1-benzyl-3-hydroxy-2-methylpyridin-4(1H)-one **4** (200 mg, 0.930 mmol), Et_3N (150 mg, 1.49 mmol) and pyridine (118 mg, 1.49 mmol) in anhydrous DCM (4 mL) was added methylcarbamic chloride (81 mg, 1.0 mmol) dropwise at 0 °C. After addition, the mixture was stirred at 25 °C for 16 h. Then the reaction was quenched with water and extracted with EtOAc (10 mL x 3). The combined organic layers were washed with brine, dried over anhydrous Na_2SO_4, filtered, and concentrated *in vacuo*. The residue was first purified by silica gel chromatography (DCM/MeOH, 0% to 30%), then further purified by prep-HPLC (Gemini 5 μm C_{18} column, 150*21.2 mm, eluting with 20% to 25% MeCN/H_2O containing 0.1% formic acid) to afford the desired

product **6** (19 mg, 7.5% yield) as a white solid: ^{1}H NMR (400 MHz, DMSO-d_6, ppm) δ 7.83 (d, *J* = 7.6 Hz, 1 H), 7.47 (d, *J* = 4.5 Hz, 1 H), 7.40 (t, *J* = 7.4 Hz, 2 H), 7.33 (d, *J* = 7.2 Hz, 1 H), 7.12 (d, *J* = 7.3 Hz, 2 H), 6.20 (d, *J* = 7.6 Hz, 1 H), 5.26 (s, 2 H), 2.61 (d, *J* = 4.6 Hz, 3 H), 2.06 (s, 3 H). LC-MS (ESI) calcd. for $C_{15}H_{17}N_2O_3$ $[M + H]^+$ m/z 273.12, found 273.10

A solution of 1-benzyl-2-methyl-4-oxo-1,4-dihydropyridin-3-yl methylcarbamate **6** (150 mg, 0.500 mmol) and 20% $Pd(OH)_2$/C (30 mg) in MeOH (5 mL) was evacuated and backfilled with hydrogen three times and then charged with hydrogen. The resulting mixture was stirred at rt for 16 h. Then the mixture was filtered through Celite. The filtrate was concentrated to give crude product. The residue was purified by prep-HPLC (Gemini 5 µm C_{18} column, 150*21.2 mm, eluting with 2% to 10% MeCN/H_2O containing 0.1% formic acid) to afford the desired product (22 mg, 21% yield) as a light grey solid: ^{1}H NMR (400 MHz, DMSO-d_6, ppm) δ 11.49 (s, 1 H), 7.54 (s, 1 H), 7.47 (d, *J* = 4.6 Hz, 1 H), 6.13 (s, 1 H), 2.62 (d, *J* = 4.6 Hz, 3 H), 2.10 (s, 3 H). LC-MS (ESI) calcd. For $C_8H_{11}N_2O_3$ $[M + H]^+$ m/z 183.07, found 183.15.

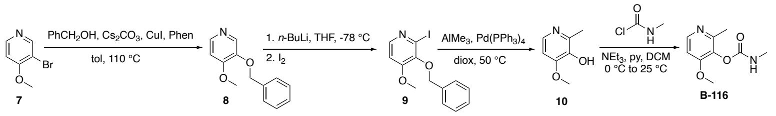

7 8 9 10 B-116

4-methoxy-2-methylpyridin-3-yl methylcarbamate (B-116): To a stirred solution of 3-bromo-4-methoxypyridine **7** (2.00 g, 10.6 mmol), Cs_2CO_3 (5.18 g, 15.9 mmol), copper(I) iodide (0.20 g, 1.1 mmol), and 1,10-phenanthroline (0.38 g, 2.1 mmol) in toluene (2 mL) was added BnOH (11.5 g, 106 mmol) in one charge under nitrogen at 25 °C. The reaction mixture was stirred at 110 °C for 24 h. The mixture was filtered, and the filtrate was concentrated *in vacuo*. The residue was purified by silica gel chromatography (EtOAc/PE, 20% to 80%) to afford the desired product **8** (700 mg, 26.0% yield) as a white solid: LC-MS (ESI) calcd. for $C_{13}H_{14}NO_2$ $[M + H]^+$ m/z 216.09, found 216.15.

To a stirred solution of **8** (700 mg, 3.25 mmol) in THF (10 mL) was added *n*-BuLi (0.4 mL, 4.88 mmol, 2.4 M in hexanes) dropwise at -78 °C under nitrogen atmosphere. The reaction mixture was stirred at -78 °C for 1 h. To the mixture was added I_2 (1.65 g, 6.50 mmol). The mixture was stirred at 25 °C for 11 h. The reaction was quenched with water (20 mL) and extracted with EtOAc (20 mL×3). The combined organic layers were washed by brine, dried over anhydrous Na_2SO_4, filtered, and concentrated *in vacuo*. The residue was purified by silica gel chromatography (EtOAc/PE, 10% to 60%) to afford the desired product **9** (350 mg, 27.0% yield) as a white solid: ^{1}H NMR (400 MHz, $CDCl_3$, ppm) δ 8.03 (d, *J* = 5.6 Hz, 1 H), 7.59 - 7.52 (m, 2 H), 7.43 - 7.30 (m, 3 H), 6.79 (d, *J* = 5.2 Hz, 1 H), 5.04 (s, 2 H), 3.90 (s, 3 H).

To a solution of 3-(benzyloxy)-2-iodo-4-methoxypyridine **9** (350 mg, 1.03 mmol) and $Pd(PPh_3)_4$ (118 mg, 0.100 mmol) in 1,4-dioxane (10 mL) was added $AlMe_3$ (2.00 mL, 2.05 mmol, 1 M in hexane) dropwise under N_2 atmosphere. Then the reaction mixture was stirred at 120 °C for 5 h. The reaction was quenched with water (10 mL) and extracted with EtOAc (10 mL×3). The combined organic layers were washed by brine, dried over anhydrous Na_2SO_4, filtered, and concentrated *in vacuo.* The residue was purified by silica gel chromatography (EtOAc/PE, 20% to 80%) to afford the desired product **10** (60 mg, 40% yield) as a white solid: LC-MS (ESI) calcd. for $C_7H_{10}NO_2$ $[M + H]^+$ m/z 140.06, found 140.20.

To a stirred solution of **10** (60 mg, 0.43 mmol) and TEA (87 mg, 0.86 mmol) in DCM (2 mL) was added *N*-methylcarbamoyl chloride (48 mg, 0.52 mmol) in one charge at 25 °C. The

reaction mixture was stirred for 2 h. Then the reaction was quenched with MeOH (2 mL) and concentrated *in vacuo.* The residue was purified by prep-TLC (MeOH/DCM, 0% to 90%) to afford the desired product **B-116** (19 mg, 20% yield) as a white solid: ^{1}H NMR (400 MHz, DMSO-d_6, ppm) δ 8.18 (d, J = 5.6 Hz, 1 H), 7.72 - 7.64 (m, 1 H), 7.00 (d, J = 5.6 Hz, 1 H), 3.81 (s, 3 H), 2.66 (d, J = 4.8 Hz, 3 H), 2.25 (s, 3 H). LC-MS (ESI) calcd. for $C_9H_{13}N_2O_3$ [M + H]$^+$ m/z 197.09, found 197.20.

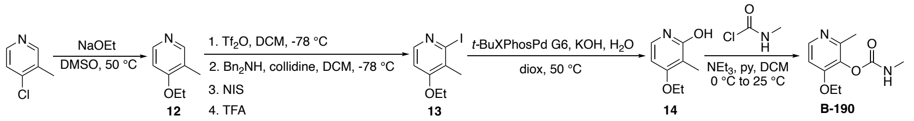

4-ethoxy-2-methylpyridin-3-yl methylcarbamate (B-190): To a solution of 4-chloro-2-methylpyridine (10.0 g, 78.4 mmol) in DMSO (100 mL) was added sodium ethoxide (26.68 g, 39.20 mmol) under N_2. The mixture was heated at 50 °C for 16 h. The reaction was washed with water and extracted with EtOAc. The combined organic layers were washed with brine, dried over anhydrous Na_2SO_4, filtered, and concentrated *in vacuo*. The residue was purified by silica gel chromatography (MeOH/DCM, 0% to 5%) to afford the desired product **12** (6.0 g, 56% yield) as a yellow oil: LC-MS (ESI) calcd. for $C_8H_{12}NO$ [M + H]$^+$ m/z 138.15, found 138.35.

A round bottom flask equipped with a stir bar was charged with the 4-ethoxy-2-methylpyridine **12** (6.00 g, 43.8 mmol) and placed under a nitrogen atmosphere. DCM (300 mL) was added, the reaction flask was cooled to -78 °C then Tf_2O (12.36 g, 43.80 mmol) was added dropwise. The reaction was stirred for 30 min before a DCM solution (1.0 M) of dibenzylamine (8.64 g, 43.8 mmol) was added, followed by collidine (5.31 g, 43.8 mmol). The reaction was stirred for a further 30 min at -78 °C. The cooling bath was removed, and the reaction was allowed to warm to 20 °C while stirring for approximately 30 min. N-iodosuccinimide (9.84 g, 43.8 mmol) was added, and then the reaction was allowed to stir at rt for 10 min. Trifluoroacetic acid (9.99 g, 87.6 mmol) was added, and the reaction was stirred at 20 °C for 2 h. The reaction was diluted with DCM and H_2O, then extracted into DCM (3 x 300 mL). The organic extract was dried over $MgSO_4$, filtered, and concentrated under vacuum. The residue was purified with flash column chromatography (PE/EtOAc, 0% to 35%) to the desired product 1**3** (1 g, 9% yield) as a yellow oil: LC-MS (ESI) calcd. for $C_8H_{11}INO$ [M + H]$^+$ m/z 263.99, found 264.00.

To a mixture of **13** (200 mg, 0.760 mmol) and tBuXPhos Pd G6 (60 mg, 0.076 mmol) in dioxane (2 mL) was added KOH (128 mg, 2.28 mmol) and H_2O (268 mg, 15.2 mmol) under N_2. The mixture was heated at 50 °C for 16 h. The reaction was diluted with water and extracted with EtOAc. The combined organic layers were washed with brine, dried over anhydrous Na_2SO_4, filtered, and concentrated *in vacuo*. The residue was purified by silica gel chromatography (MeOH/DCM, 0% to 5%) to afford the desired product **14** (100 mg, 85.0% yield) as a yellow oil: LC-MS (ESI) calcd. for $C_8H_{12}NO_2$ [M + H]$^+$ m/z 154.08, found 154.25.

To a solution of **14** (100 mg, 0.650 mmol) in DCM (1 mL) was added N-methylcarbamoyl chloride (92 mg, 0.98 mmol) and triethylamine (198 mg, 1.96 mmol) slowly. The mixture was stirred at 20 °C for 2 h. The mixture was concentrated under vacuum. The crude product was purified by prep-TLC (EtOAc/PE, 1/3) to obtain the desired product **B-190** (11 mg, 8.0% yield) as a white solid: ^{1}H NMR (400 MHz, DMSO-d_6) δ 8.14 (d, J = 5.5 Hz, 1 H), 7.75-7.63 (m, 1 H), 6.98

(d, J = 5.5 Hz, 1 H), 4.10 (q, J = 6.8 Hz, 2 H), 2.66 (d, J = 4.3 Hz, 3 H), 2.24 (s, 3 H), 1.28 (t, J = 6.8 Hz, 3 H). LCMS (ESI) calcd. for $C_{10}H_{15}N_2O_3$ $[M + H]^+$ m/z 211.17, found 211.15.

Compounds **B-201, 217, and 219** were prepared via a similar route and their spectroscopic data are presented below.

4-(benzyloxy)-2-methylpyridin-3-yl methylcarbamate (B-201): ^{1}H NMR (400 MHz, DMSO-d_6, ppm) δ 8.16 (d, J = 5.6 Hz, 1 H), 7.73 (d, J = 4.4 Hz, 1 H), 7.43-7.36 (m, 4 H), 7.34 (dd, J = 5.6, 2.8 Hz, 1 H), 7.05 (d, J = 5.6 Hz, 1H), 5.21 (s, 2 H), 2.67 (d, J = 4.5 Hz, 3 H), 2.28 (s, 3 H). LC-MS (ESI) calcd. for $C_{15}H_{17}N_2O_3$ $[M + H]^+$ m/z 273.12, found 273.10.

4-isopropoxy-2-methylpyridin-3-yl methylcarbamate (B-217): ^{1}H NMR (400 MHz, DMSO-d_6, ppm) δ 8.12 (d, J = 5.6 Hz, 1 H), 7.66 (d, J = 4.4 Hz, 1 H), 7.00 (d, J = 5.7 Hz, 1 H), 4.69 (p, J = 6.0 Hz, 1 H), 2.66 (d, J = 4.6 Hz, 3 H), 2.24 (s, 3 H), 1.24 (d, J = 6.0 Hz, 6 H). LC-MS (ESI) calcd. for $C_{11}H_{18}N_2O_3$ $[M + H]^+$ m/z 225.12, found 225.10.

4-(cyclohexylmethoxy)-2-methylpyridin-3-yl methylcarbamate (B-219): ^{1}H NMR (400 MHz, DMSO-d_6, ppm) δ 8.13 (d, J = 5.6 Hz, 1 H), 7.70 (d, J = 4.5 Hz, 1 H), 6.97 (d, J = 5.6 Hz, 1 H), 3.84 (d, J = 5.9 Hz, 2 H), 2.65 (d, J = 4.6 Hz, 3 H), 2.25 (s, 3 H), 1.76-1.61 (m, 5 H), 1.27-0.98 (m, 6 H). LC-MS (ESI) calcd. for $C_{15}H_{23}N_2O_3$ $[M + H]^+$ m/z 279.16, found 279.15.

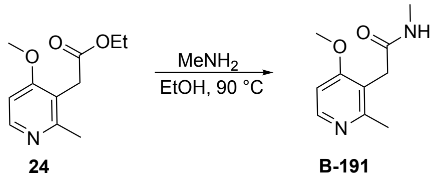

2-(4-methoxy-2-methylpyridin-3-yl)-N-methylacetamide (B-191): A solution of ethyl 2-(4-methoxy-2-methylpyridin-3-yl)acetate **24** (100 mg, 0.470 mmol) in 1N $MeNH_2$ (EtOH solution; 3 mL) was heated at 90 °C for 48 h in a sealed tube. Then, the mixture was concentrated under reduced pressure. The residue was directly purified by prep-HPLC (Gemini 5 μm C_{18} column, 150*21.2 mm, eluting with 0% to 30% MeCN/H_2O containing 0.1% NH_4OH) to give the desired product (12.15 mg, 12.43 % yield): ^{1}H NMR (400 MHz, DMSO-d_6, ppm) δ 8.25 (d, J = 5.8 Hz, 1 H), 7.77-7.71 (m, 1 H), 6.94 (d, J = 5.9 Hz, 1 H), 3.82 (s, 3 H), 3.46 (s, 2 H), 2.56 (d, J = 4.6 Hz, 3 H), 2.38 (s, 3 H). LC-MS (ESI) calcd. for $C_{10}H_{15}N_2O_2$ $[M + H]^+$ m/z 195.11, found 195.00.

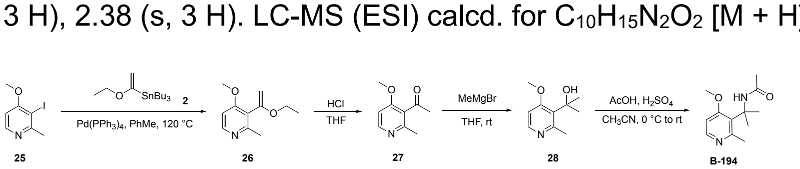

N-(2-(4-methoxy-2-methylpyridin-3-yl)propan-2-yl)acetamide (B-194): To a solution of 3-iodo-4-methoxy-2-methylpyridine **25** (1.5 g, 6.0 mmol) in PhMe (15 mL) was added tri-*n*-butyl(1-ethoxyethenyl)stannane (435 mg, 12.0 mmol) and $Pd(PPh_3)_4$ (696 mg, 0.6 mmol) slowly under N_2. The mixture was heated at 120 °C for 16 h. Upon completion of the reaction, the mixture was concentrated *in vacuo*, and purified by silica gel column chromatography (eluting with EtOAc/PE,

0% to 30%) to give the desired product **26** (1.1 g, 93% yield) as a yellow solid: LC-MS (ESI) calcd. for $C_{11}H_{16}NO_2$ [M + H]$^+$ m/z 194.11, found 194.10.

To a solution of **26** (1.1 g, 5.6 mmol) in THF (2 mL) was added 2M HCl (2 mL). The mixture was stirred at 20 °C for 2 h. Then the solution was adjusted to pH 6-7 with saturated aqueous $NaHCO_3$ and extracted with EtOAc. The combined organic layers were washed by brine, dried over anhydrous Na_2SO_4, filtered, and concentrated *in vacuo*. The residue was purified by silica gel chromatography (DCM/MeOH, 0% to 5%) to give the desired product **27** (630 mg, 65.0% yield) as a yellow solid: LC-MS (ESI) calcd. for $C_9H_{12}NO_2$ [M + H]$^+$ m/z 166.08, found 166.25.

To a solution of **27** (500 mg, 3.03 mmol) in THF (5 mL) was added MeMgBr (1M in THF, 6.05 mmol) slowly under N_2. The mixture was stirred at 20 °C for 16 h. Then the reaction was quenched with saturated aqueous NH_4Cl and extracted with EtOAc. The combined organic layers were washed with brine, dried over anhydrous Na_2SO_4, filtered, and concentrated *in vacuo*. The residue was purified by silica gel chromatography (DCM/MeOH, 0% to 5%) to give the desired product **28** (350 mg, 64.0% yield) as a yellow solid: LC-MS (ESI) calcd. for $C_{10}H_{16}NO_2$ [M + H]$^+$ m/z 182.11, found 182.30.

To a solution of **28** (350 mg, 1.9- mmol) in MeCN (4 mL) was added AcOH (1.5- g, 25.1 mmol) and sulfuric acid (2.65 g, 27.0 mmol) slowly at 0 °C. The mixture was stirred at 20 °C for 2 h. Upon completion of the reaction, the solution was adjusted to pH 6-7 with 6N NaOH and extracted with EtOAc. The combined organic layers were concentrated *in vacuo* and purified by prep-HPLC (Gemini 5 μm C_{18} column, 150*21.2 mm, eluting with 0% to 30% $MeCN/H_2O$ containing 0.1% NH_4OH) to afford the desired product **B-194** (34 mg, 8.0% yield, white solid): ^1H NMR (400 MHz, Methanol-d_4) δ 8.34 (s, 1 H), 8.23 (d, J = 6.3 Hz, 1 H), 7.16 (d, J = 6.3 Hz, 1 H), 3.98 (s, 3 H), 2.67 (s, 3 H), 1.91 (s, 3 H), 1.77 (s, 6 H). LC-MS (ESI) calcd. for $C_{12}H_{19}N_2O_2$ [M + H]$^+$ m/z 223.14, found 223.10.

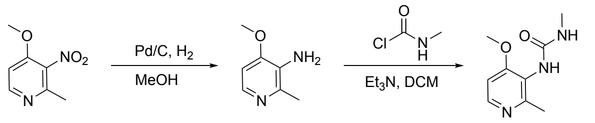

29 30 B-195

1-(4-methoxy-2-methylpyridin-3-yl)-3-methylurea (B-195): A solution of 4-methoxy-2-methyl-3-nitropyridine **29** (100 mg, 0.590 mmol) and 10% Pd/C (20 mg) in MeOH (2 mL) was evacuated and backfilled with hydrogen three times and then charged with hydrogen. The resulting mixture was stirred at rt for 16 h. Then the mixture was filtered through Celite. The filtrate was concentrated to give the desired product **30** (50 mg, 61% yield) as a yellow solid: LC-MS (ESI) calcd. for $C_7H_{11}N_2O$ [M + H]$^+$ m/z 139.08, found 139.10.

To a solution of **30** (50 mg, 0.36 mmol) in DCM (1 mL) was added methylcarbamic chloride (50 mg, 0.54 mmol) and triethylamine (109 mg, 1.08 mmol) slowly at 0 °C under nitrogen. The mixture was warmed to 25 °C for 2 h4. Upon the reaction was completed, the mixture was concentrated *in vacuo*. The residue was directly purified by prep-HPLC (Gemini 5 μm C_{18} column, 150*21.2 mm, eluting with 0% to 20% $MeCN/H_2O$ containing 0.1% formic acid) to afford the desired product **B-195** (27.16 mg, 38.44% yield) as a white solid: ^1H NMR (400 MHz, DMSO-d_6, ppm) δ 8.15 (d, J = 5.6 Hz, 1 H), 7.39 (s, 1 H), 6.90 (d, J = 5.6 Hz, 1 H), 6.08 (d, J = 4.5 Hz, 1 H),

3.80 (s, 3 H), 2.60 (d, *J* = 4.6 Hz, 3 H), 2.28 (s, 3 H). LC-MS (ESI) calcd. for C9H14N3O2 $[M + H]^+$ m/z 196.10, found 196.05.

Appendix Supplementary Note S1

The distribution of positive and negative control signals across all plates screened was investigated and the analysis presented in the below figure. The histograms quantify the proportion of HIV control models in control conditions (DMSO or Lopinavir) as a distribution across all plates. To compute this, barcoded strain read counts were first normalized by the total read counts in each well. Then, for each plate, the mean of well-normalized HIV read counts was calculated across all barcodes in a control condition (DMSO or Lopinavir). Repeating this for all plates produced the below histograms.

By comparing HIV in Lopinavir and HIV in DMSO, the z-prime metric (Zhang et al., 1999) was computed as 0.091 with the positive control mean ± std of 0.028 ± 0.004 and negative control of 0.0067 ± 0.0030 well-normalized read counts. Thus, although Lopinavir properly functioned as a positive control, the assay signal was weak, which necessitated the development of our novel hit calling methods to achieve good discriminative capabilities.

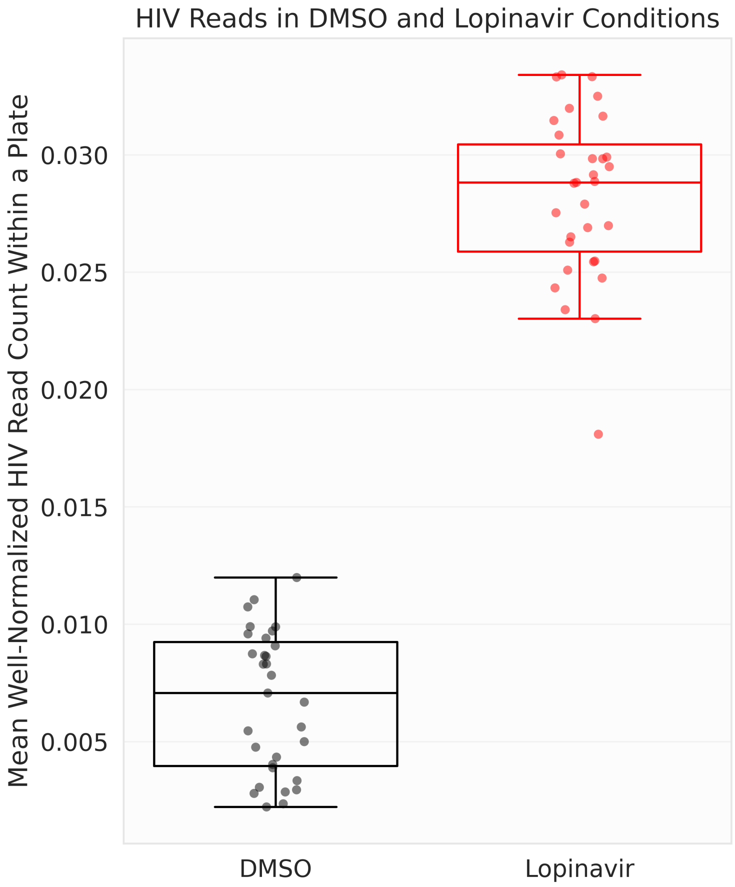

We investigated the effects of log transformation on our data and compared the z-prime metric compared across different transformations. Starting with raw read counts, log transformation increases the z-prime metric from -1.4 to -1.0. By contrast, well-normalization increases the z-prime metric from -1.4 to 0.091. Combining these normalizations, however, does not further increase z-prime; log transformation along with well-normalization brings the z-metric down to -0.52. Although the z-prime metric may suggest that the assay cannot precisely distinguish viral inhibitors, the robust computational pipeline that leverages our built-in controls was developed to overcome this challenge.

Zhang JH, Chung TD, Oldenburg KR. A simple statistical parameter for use in evaluation and validation of high throughput screening assays. Journal of biomolecular screening. 1999 Apr;4(2):67-73. doi: 10.1177/108705719900400206

Appendix Supplementary Note S2

The costs associated with sequencing, excluding those related to compound acquisition and outgrowth (which are comparable between the current and conventional methods), are outlined below:

Library preparation:
Taq polymerase + buffer: $0.02/well
dNTPs: $0.02/well
Forward and Reverse Primers: $0.01/well
PCR plate: $0.07/well
PCR plate seal: $0.01/well

Library sequencing:
Illumina NextSeq 550, 75bp single end sequencing: $0.80/well

Total sequencing costs per compound screened (including PCR technical replicates): $1.06/well

Further cost reductions are anticipated due to the increased availability of partial sequencing runs at shared facilities, the development of more cost-effective sequencing platforms (e.g., NovaSeq), and the ongoing emergence of competitors in the short-read sequencing market. These factors are expected to drive down sequencing costs even further, enhancing the overall cost-efficiency of our approach.
